# Comparable Outcomes in Acquired Severe Aplastic Anemia Patients With Haploidentical Donor or Matched Related Donor Transplantation: A Retrospective Single-Center Experience

**DOI:** 10.3389/fmed.2021.807527

**Published:** 2022-01-24

**Authors:** QingYun Wang, HanYun Ren, ZeYin Liang, Wei Liu, Yue Yin, QingYa Wang, Qian Wang, YuHua Sun, WeiLin Xu, ZhiXiang Qiu, JinPing Ou, Na Han, Jing Wang, YuJun Dong, Yuan Li

**Affiliations:** Department of Hematology, Peking University First Hospital, Beijing, China

**Keywords:** severe aplastic anemia, hematopoietic stem cell transplantation, haploidentical donor, matched related sibling donor, survival

## Abstract

Clinical data of patients with severe aplastic anemia (SAA) were retrospectively analyzed to evaluate the outcomes of haploidentical hematopoietic stem cell transplantation (HID-HSCT) with matched related sibling hematopoietic stem cell transplantation (MSD-HSCT) in complications and survivals. Thirty consecutive patients were enrolled in the study with a median follow-up of 50 months (range 4, 141), and the median age of the patients was 21 years (range 3, 49). All the patients achieved myeloid engraftment in the two cohorts. The cumulative incidences of platelet engraftment were 95.5 and 100% in HID cohort and MSD cohort, respectively. The median time for neutrophil and platelet recovery was 11 (range 9, 19) and 15 (range 10, 25) days in HID cohort, and 12 (range 10, 19) and 14 (range 8, 25) days in MSD cohort. The cumulative incidences of grade II–IV and grade III–IV acute graft vs. host disease (aGvHD) in HID cohort and in MSD cohort were 18.9 vs. 14.3% (*p* = 0.77) and 10.5 vs. 0% (*p* = 0.42), respectively. The cumulative incidences of chronic graft vs. host disease (cGvHD) was 22.7% in HID cohort and 25.5% in MSD cohort (*p* = 0.868). The 5-year overall survival (OS) rates and 5-year failure-free survival (FFS) rates in HID cohort and MSD cohort were 85.1 vs. 87.5% (*p* = 0.858), 80.3 vs. 87.5% (*p* = 0.635), respectively. The median time to achieve engraftment, cumulative incidence of aGvHD and cGvHD, and the 5-year OS and FFS rates were not significantly different between the two cohorts. We suggest that HID-HSCT might be a safety and effective option for SAA patients without a matched donor.

## Introduction

Severe aplastic anemia (SAA) is a potentially fatal disorder due to bone marrow failure. Immunosuppressive therapy (IST) and allogeneic hematopoietic stem cell transplantation (allo-HSCT) are principle treatment modalities (1, 2). However, patients are not cured by IST, and some patients are at risk of lack of response, relapse, and clonal evolution (1). According to the treatment guidelines, HLA-matched sibling donor (MSD) HSCT is recommended for the patients with SAA younger than 40 years as first-line therapy (1, 3–6). Considering the transplantation-related mortality (TRM) rate and long-time survival rate, haploidentical donor (HID) HSCT is recommended only when IST has failed and no MSD or unrelated relative donor (URD) is available (1). In recent years, with the improvement of conditioning regimen and the development of graft vs. host disease (GvHD) prevention and supportive care, TRM of HID-HSCT has significantly declined. Recent reports showed that the results of HID-HSCT have been more promising particular in younger patients (7–9). Considered for easy donor availability, HID-HSCT has been regarded as an alternative treatment modality for patients without MSD or URD. However, few reports have compared the outcomes between HID-HSCT and MSD-HSCT. To further evaluate the safety and efficiency of HID-HSCT for the treatment of SAA, we retrospectively analyzed the statistics of 30 patients who underwent allo-HSCT in our center.

## Patients and Methods

### Participants

A total of 30 consecutive patients with SAA who underwent allo-HSCT between March 2010 and June 2021 at Peking University First Hospital were enrolled in this research. Diagnosis of SAA or VSAA was based on the Camitta's criteria (10). Patients with clonal evolution were excluded from this study. None of the patients had chromosomal abnormalities. Among the patients enrolled in this study, 17 were men and 13 were women. Twenty-two patients received HID-HSCT, and eight patients received MSD-HSCT. The median age was 21 (range 3, 49) years. The median interval from diagnosis to transplantation was 3 (range 1, 85) months. We followed the patients until September 30, 2021, with a median follow-up of 50 (range 4, 141) months.

### Conditioning Regimen

The conditioning regimen consisted of the following: busulfan (Bu) 0.8 mg/kg four times daily intravenously (iv.) for 2 days, cyclophosphamide (Cy) 50 mg/kg daily iv. for 4 days (Bu/Cy); fludarabine (Flu) 25 mg/m^2^ daily iv. for 5 days, busulfan 0.8 mg/kg four times daily iv. for 2 days, cyclophosphamide 25 mg/kg daily iv. for 4 days (Flu-Bu/Cy); cyclophosphamide 50 mg/kg daily iv. for 4 days, fludarabine 25 mg/m^2^ daily iv. for 5 days (Cy/Flu); cyclophosphamide 50 mg/kg daily iv. for 4 days (Cy). All the patients received rabbit antithymocyte globulin (rATG) 2.5 mg/kg daily iv. for 4 days. The conditioning regimen used in HID and MSD cohort is listed in Table 1.

**Table 1:** Clinical characteristics of the patients.

	**HID**	**MSD**
Number	22	8
Gender (male/female)	13/9	4/4
Age	18.5 (3–49)	26.5 (10–47)
Interval from diagnosis to HSCT (months)	3.5 (1–85)	2.5 (1–50)
Previous IST with ATG, no. (%)	3 (14.3%)	0
Donor age	37 (12–54)	27 (18–41)
Donor gender		
Male	12	4
Female	10	4
Donor type		
Parent	14	–
Child	3	–
Sibling	5	8
ABO blood type (match/mismatch)	11/11	3/5
Conditioning regimen		
Cy/Flu	7	6
Cy	0	2
Bu/Cy/Flu	11	0
Bu/Cy	4	0
Stem cell source		
BM+PB	18	6
PB	4	2
MNC (×10^8^/kg)	11.88 (4.94–16.7)	8.96 (5.79–12.81)
CD34^+^ cells (×10^6^/kg)	4.51 (1.80–9.32)	5.28 (2.27–7.85)
DSA		
MFI 2,000–10,000	1	–
MFI 500–2,000	2	–
MFI <500	5	–
NA	14	–

*HID, haploidentical donor; MSD, matched sibling donor; Cy, cyclophosphamide; Flu, fludarabine; Bu, busulfan; rATG, rabbit antithymocyte globulin; BM, bone marrow; PB, peripheral blood; MNC, mononuclear cell; DSA, donor-specific anti-HLA antibodies; MFI, mean fluorescence intensity; NA, not available*.

### Prophylaxis and Treatment of GvHD

Graft vs. host disease prophylaxis consisted of cyclosporin A (CsA), mycophenolate mofetil (MMF), and methotrexate (MTX). CsA was administered iv. at a dosage of 5 mg/kg daily from day −6 to the time of bowel function recovery, and then switched to oral administration. CsA concentration was aimed to sustain from 200 to 250 ng/ml. MMF was administered orally at a dosage of 500 mg two times a day from the start of conditioning up to days +30 post-HSCT. MTX was administered at a dose of 10–15 mg/m^2^ iv. on days +1, +3, +5, +11 after transplantation. First-line therapy of grade II–IV, aGvHD was methylprednisolone 1–2 mg/kg daily. Patients refractory to steroid therapy received tacrolimus, anti-CD25 monoclonal antibody, etc. Immunosuppressants were adjusted according to the site and severity of cGvHD. If the patients had extensive cGvHD and were refractory to normal immunosuppressants, anti-CD25 monoclonal antibody, rituximab or ruxolitinib was administered. Acute graft vs. host disease (aGvHD) was diagnosed and graded according to Seattle criteria (11). Chronic graft vs. host disease (cGvHD) of patients survived to days +100 post-HSCT was diagnosed and graded according to the criteria by National Institutes of Health (NIH) (12).

### Stem Cell Mobilization, Collection, and Type of Transplantation

Donors received granulocyte colony stimulating factor (G-CSF) 5 μg/kg subcutaneously every 12 h for 5 consecutive days. Bone marrow stem cells were collected on the 4th day, and peripheral blood stem cell (PBSC) harvesting was carried out on the 5th day after G-CSF treatment.

Twenty-four of the patients received bone marrow plus PBSCs, and six received PBSCs only (donors of the 6 patients refused to donate bone marrow stem cells). The median amount of infused mononuclear cells (MNCs) and CD34^+^ cells was 10.68 (4.94–16.70) × 10^8^/kg, 4.62 (1.80–9.32) × 10^6^/kg, respectively.

### Supportive Care

Appropriate antibiotics were used to prevent microbial infection. Ganciclovir was administered iv. between days −9 and −2 during conditioning to prevent cytomegalovirus (CMV) infection. Voriconazole was used for fungus prophylaxis treatment. Acyclovir and SMZ/TMP were administered orally to prevent herpes virus infection and pneumocystis carinii pneumonitis (PCP) from days +1 to 6 months post-HSCT. G-CSF was administered subcutaneously from days +6 until neutrophil engraftment. Thrombopoietin (TPO) was administered subcutaneously from day +1 until platelet engraftment. Irradiated blood products were given for all the patients. CMV and Epstein-Barr virus (EBV) PCR were monitored once a week.

### Hematopoietic Restruction and Definition

Neutrophil engraftment was defined as absolute neutrophil count ≥0.5 × 10^9^/L for 3 consecutive days. Platelet engraftment was defined as absolute platelet count ≥20 × 10^9^/L for 7 consecutive days without platelet transfusion. Full donor chimerism was defined as hematopoietic cells from donor in the bone marrow >95%. Primary graft failure (PGF) was defined as neutrophil or platelet was under the level of engraftment by days +28. Secondary graft failure (SGF) was defined as neutrophil <0.5 × 10^9^/L or platelet <20 × 10^9^/L with low donor chimerism, or absence of donor chimerism (<5%) with the exception of relapse (13). Overall survival (OS) was defined as the time from the day of HSCT to death or the most recent follow-up. Failure-free survival (FFS) was defined as the time from the day of HSCT to death or graft failure (GF).

### Statistical Analysis

Between-group differences were assessed using the chi-square test for categorical variables and *t*-test for continuous variables. The cumulative incidence of engraftment, aGvHD, cGvHD, CMV, and EBV infection was estimated considering the competing risks. OS and FFS were calculated by Kaplan–Meier method and compared using the Log-rank test. The values of *p* were two tailed, and *p* < 0.05 was considered indicative of statistical significance. The analyses were performed with SPSS 21.0 (SPSS Inc.) and R statistical software (Comprehensive R Archive Network, TUNA, Tsinghua University, China).

## Results

### Clinical Characteristics

Thirty patients of SAA were enrolled in our research. There were 22 patients in HID cohort and 8 patients in the MSD cohort. The traits of patients' age, interval from diagnosis to HSCT, donor age, conditioning regimen, ABO blood type, amount of MNCs, and CD34^+^ cells were matched. In HID cohort, nineteen patients received only CsA therapy as IST before HSCT, and 3 patients received CsA plus rATG as IST before HSCT. In MSD cohort, all the patients received CsA therapy before HSCT, and no patient received ATG. Donor-specific anti-HLA antibodies (DSA) have been detected in patients who received HID-HSCT since 2018, so data of 8 patients in HID cohort were acquired. Clinical characteristics of the patients are listed in Table 1.

### Hematopoietic Reconstruction

All of the 30 patients achieved myeloid engraftment (100%). In HID cohort, the median time for neutrophil recovery was 11 (range 9, 19) days. Tween-one patients achieved platelet engraftment with median time for recovery of 15 (range 10, 25) days. The cumulative incidence of platelet engraftment was 95.5%. In MSD cohort, the median time for neutrophil recovery was 12 (range 10, 19) days. All the patients achieved platelet engraftment with median time for recovery of 14 (range 8, 25) days. No significant differences were observed in neutrophil and platelet recovery time (*p* = 0.522 and 0.430, respectively). No significant differences were found in the cumulative incidence of platelet engraftment between the two cohorts (*p* = 0.546).

One patient in HID cohort experienced PGF, and platelet achieved engraftment at 107 days post-HSCT after donor PBSCs infusion. One patient in HID cohort experienced SGF after 4 months post-HSCT. The blood cell count and donor chimerism of the patient recovered to normal levels after donor PBSCs infusion. One patient in MSD cohort was diagnosed with cerebral hemorrhage after 6 months post-HSCT with low platelet count, and we considered the patient might experience SGF. The characteristics of the three patients experienced GF were listed in Table 2.

**Table 2:** Characteristics of patients with graft failure.

**Patient**	**No. 1**	**No. 2**	**No. 3**
Graft failure	PGF	SGF	SGF
Interval from diagnosis to HSCT (months)	3	39	3
Stem cell source	BM+PB	BM+PB	PB
Donor	Father (HID)	Father (HID)	Brother (MSD)
MNCs (×10^8^/kg)	14.31	5.13	8.76
CD34^+^ cells (×10^6^/kg)	3.09	2.28	5.29
DSA	NA	NA	NA

*PGF, primary graft failure; SGF, secondary graft failure; BM, bone marrow; PB, peripheral blood; MNC, mononuclear cell; DSA, donor-specific anti-HLA antibodies; NA, not available*.

### Graft vs. Host Disease

Eight patients experienced aGvHD within 100 days after HSCT, and the cumulative incidence was 26.7%. The cumulative incidence of grade II–IV aGvHD and grade III–IV were 17.7 and 8.0%, respectively. Seven patients experienced cGvHD (extensive, *n* = 2; local, *n* = 5). The cumulative incidence of cGvHD in 5 years was 20.7%.

**Figure 1:**
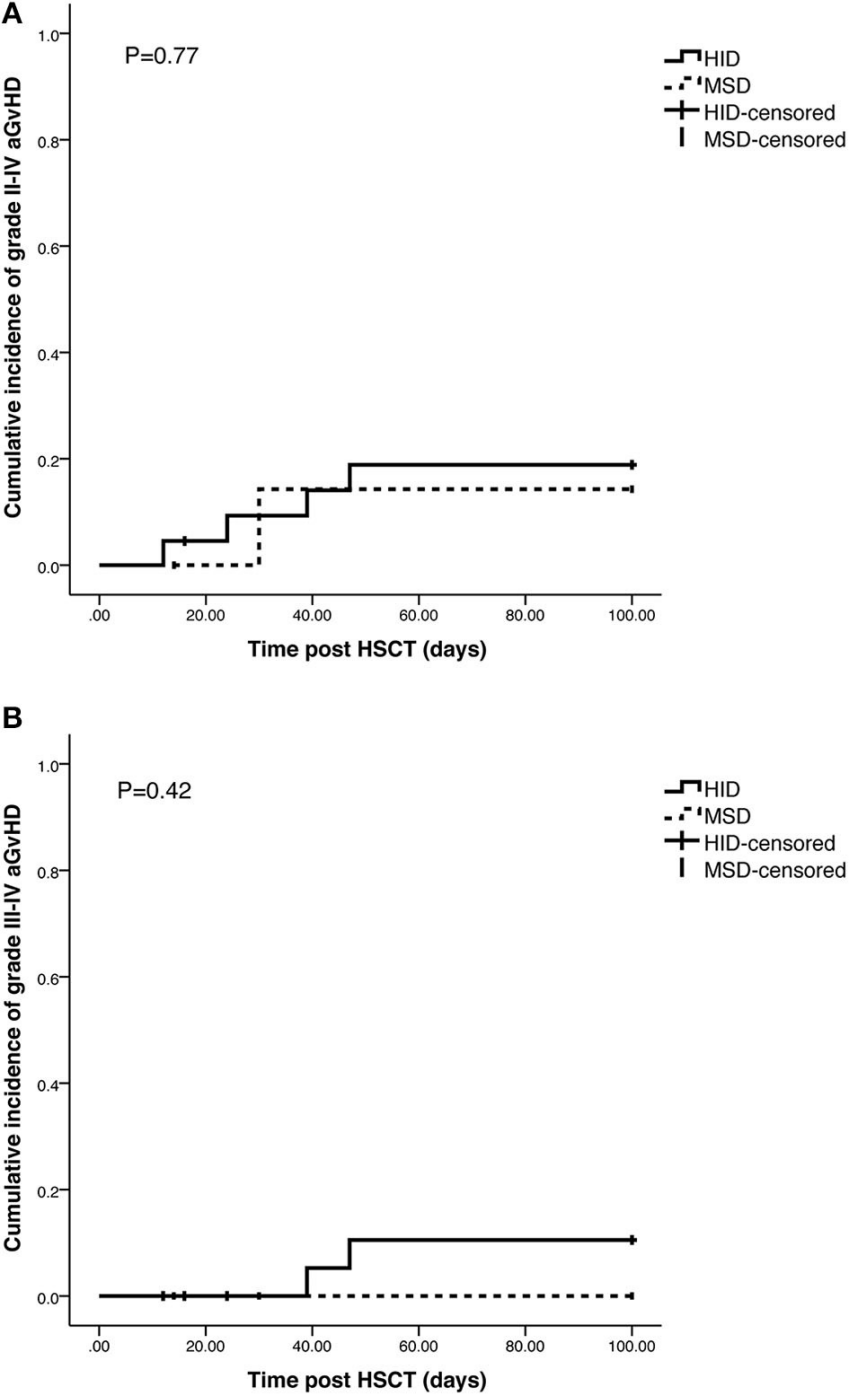
Cumulative incidence of acute graft vs. host disease (aGvHD). **(A)** Cumulative incidence of Grade II–IV aGvHD in haploidentical donor (HID) cohort and in matched sibling donor (MSD) cohort. **(B)** Cumulative incidence of Grade III–IV aGvHD in HID cohort and in MSD cohort.

**Figure 2:**
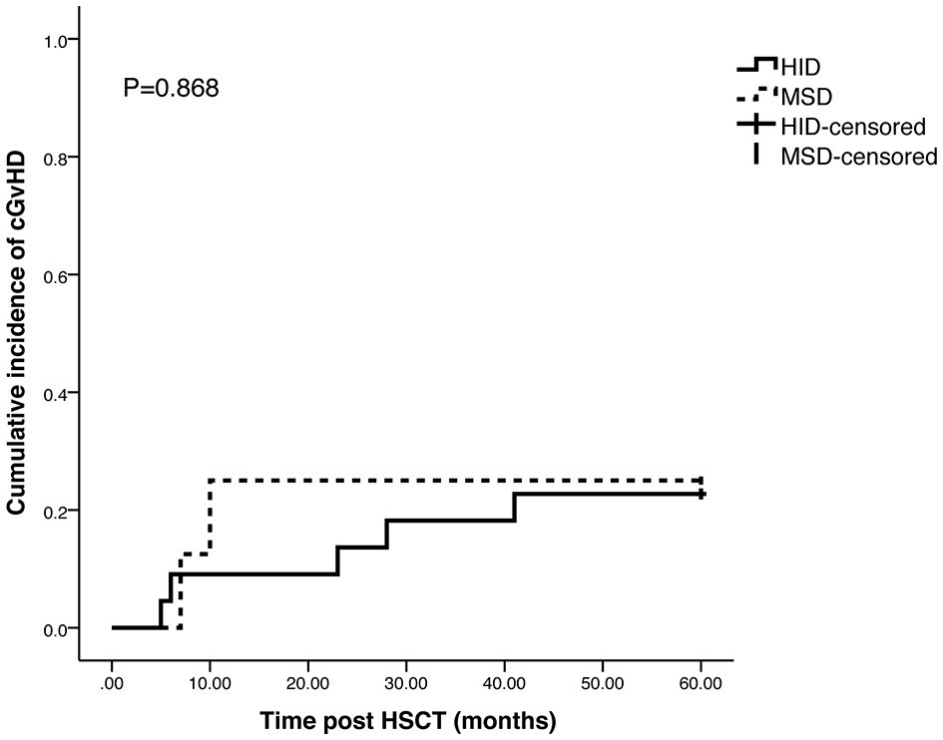
Cumulative incidence of chronic GvHD (cGvHD) in HID cohort and MSD cohort.

The cumulative incidences of grade II–IV and grade III–IV aGvHD in HID cohort and MSD cohort were 18.9 vs. 14.3% (*p* = 0.77) and 10.5 vs. 0.0% (*p* = 0.42), respectively, and no significant differences were observed between the 2 cohorts (Figure 1). The cumulative incidences of cGvHD at 5 years were 22.7% in HID cohort and 25.5% in MSD cohort (*p* = 0.868) (Figure 2).

### Infection

**Figure 3:**
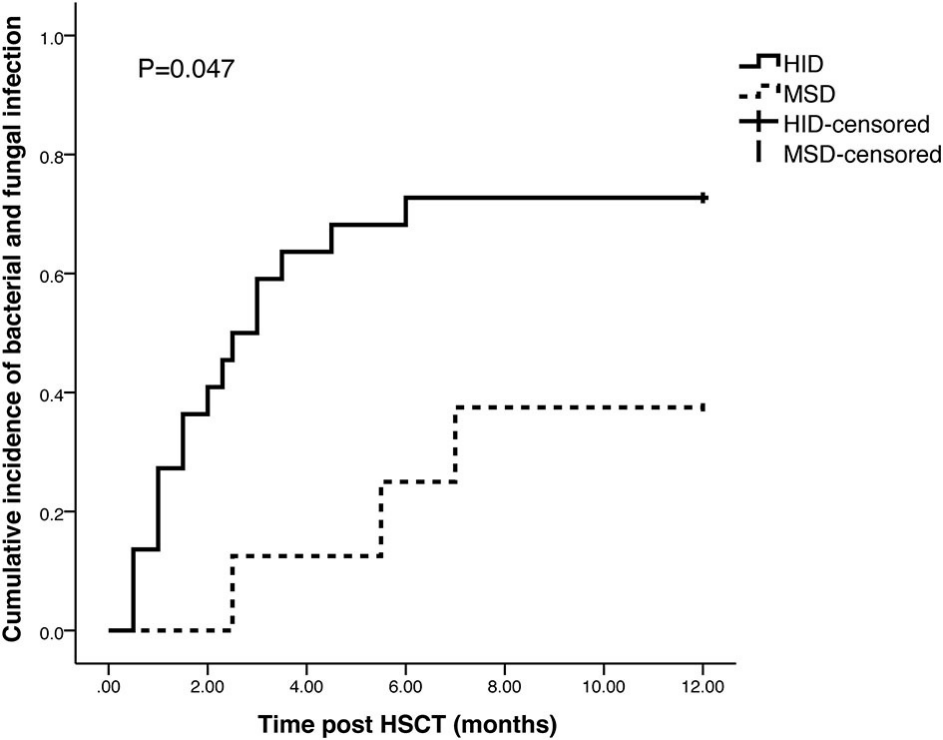
Cumulative incidence of bacterial and fungal infection in HID and MSD cohort.

Nineteen patients experienced infections in 1 year after HSCT, such as bacterial (*n* = 14), fungal (*n* = 4), and PCP (*n* = 1). The cumulative incidence of infection was 72.7% in HID cohort and 37.5% in MSD cohort with a significant difference observed (*p* = 0.047) (Figure 3).

**Figure 4:**
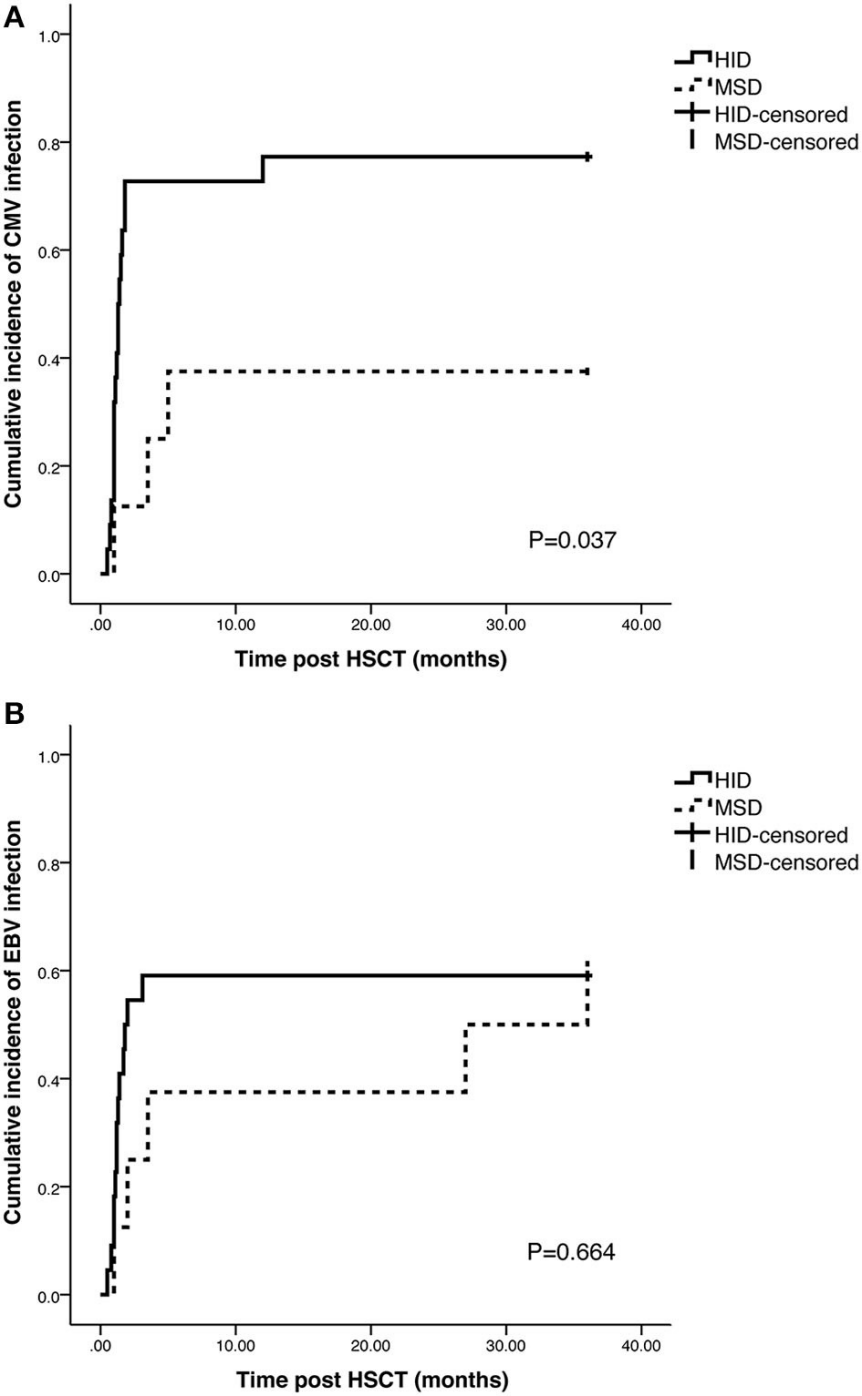
Cumulative incidence of cytomegalovirus (CMV) and Epstein-Barr virus (EBV) infection in HID and MSD cohort. **(A)** Cumulative incidence of CMV infection. **(B)** Cumulative incidence of EBV infection.

Twenty patients experienced CMV infection in 3 years after HSCT. The cumulative incidence of CMV infection in HID cohort was significantly higher than that in MSD cohort (77.3 vs. 37.5%, *p* = 0.037) (Figure 4A). In HID cohort, five patients progressed to CMV end-organ disease such as (*n* = 3), gastroenteritis (*n* = 1), and pneumonia (*n* = 1). None of patients in MSD cohort progressed to CMV end-organ disease. EBV infection was observed in 12 patients in 3 years after HSCT. The cumulative incidence of EBV infection was 59.1% in HID cohort and 62.5% in MSD cohort, and no significant difference was observed (*p* = 0.664) (Figure 4B). Four patients progressed to post-transplant lymphoproliferative disorders, and all of them recovered after the administration of rituximab.

### Survival Analysis

**Figure 5:**
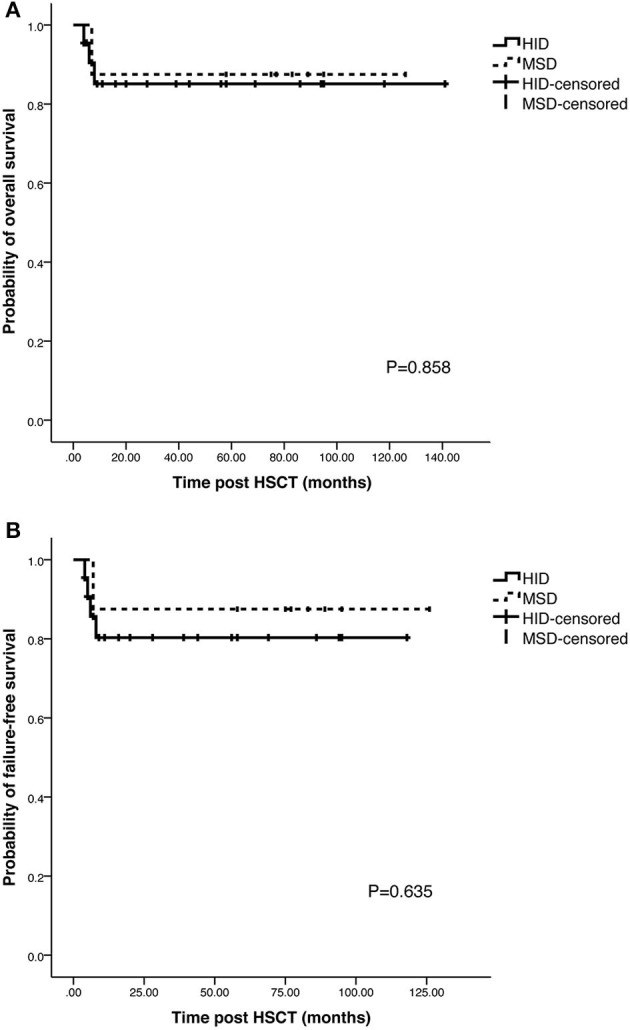
Probability of overall survival and failure-free survival in HID and MSD cohort. **(A)** 5-year OS in HID and MSD cohort. **(B)** 5-year FFS in HID and MSD cohort.

The probability of OS at 5 years was 85.1% in HID cohort and 87.5% in MSD cohort. The probability of FFS at 5 years was 80.3% in HID cohort and 87.5% in MSD cohort. No significant difference was found in 5-year OS (*p* = 0.858) and 5-year FFS (*p* = 0.635) (Figure 5). The reasons of death included PCP infection (*n* = 1, 7 months after HSCT), bacterial sepsis (*n* = 1, 4 months after HSCT), cerebral hemorrhage (*n* = 1, 6 months after HSCT), and bacterial pneumonia (*n* = 1, 4 months after HSCT).

Risk factors were analyzed by COX regression models. Factors such as patient age, donor age, gender of patient and donor, interval from diagnosis to HSCT, previous rATG before HSCT, ABO blood type, amount of MNCs and CD34^+^ cells, conditioning regimen, stem cell source, and CMV infection had no influence on OS or FFS.

## Discussion

Immunosuppressive therapy and HLA-MSD HSCT are principle treatment modalities for acquired SAA. Studies showed that OS rate was similar between the two modalities. However, the event-free survival and 10-year survival rate in MSD-HSCT group were superior to those in IST group (14, 15). MSD-HSCT has been recommended as first-line treatment modality for patients under 50 years in the British Guideline 2015 for SAA (2, 5). In the absence of MSD, URD-HSCT is recommended. But only 25–30% patients could have MSD (5, 16), and searching URD usually takes several months that may not suitable for the patient of SAA in urgent conditions. HID-HSCT has provided an alternative modality for these patients. In China, HID-HSCT has been recommended as first-line treatment modality for children (17).

The OS rate for the patients of SAA received allo-HSCT has been above 70% (6), but only 50% in patients whose age over 40 years (18). Zeng et al. (16) retrospectively analyzed SAA patients who received MSD or HID transplantation, the results showed that 5-year OS rates were 76.5 and 72% in MDS and HID cohort with no significant differences observed. In the subgroup analysis, they found that OS rate of patients whose age over 40 years in HID cohort was significantly inferior to those age range from 20 to 40 years, with 1-year OS rate 37.5 and 78.5%, respectively. In MSD cohort, the OS rates of the two subgroups were similar. Similar results were reported elsewhere (1, 19). Zhang et al. (7) analyzed the outcomes of the patients with SAA over 40 years who underwent HSCT. The 3-year OS rate was 86.7% in HID-HSCT cohort and 92.1% in MSD-HSCT cohort, and the 3-year FFS rate was 86.7 and 92.1%, with no significant differences observed. The survival outcomes were superior to previously report. In our study, 5-year OS rates and 5-year FFS rates were 85.1 vs. 87.5% (*p* = 0.858) and 80.3 vs. 87.5% (*p* = 0.635) in HID cohort and MSD cohort, respectively. The survival outcomes had no significant differences between the two cohorts. However, there were only 4 patients whose age over 40 years in our study; we did not classify the patients into subgroups by age to analyze the survival outcome. Factors affecting OS rate include physical performance (ECOG ≥ 2), age and red blood cell transfusions before HSCT (7, 16), but we did not find these factors had adversely association with OS in our research due to limited number of patients and loss of previous data of some patients when they came to our hospital.

In our study, the cumulative incidence of engraftment and blood cell recovery time were similar in HID and MSD cohort, which was consistent with most reports (7, 8, 16, 20, 21). Liu et al. (22) reported that TPO treatment promoted human hematopoietic stem and progenitor cell homing and subsequent engraftment to the BM in mice. Meanwhile, TPO treatment to the patients with SAA after HSCT significantly improved platelet engraftment, and the mean volume of transfusion (both platelet and red blood cell) was reduced. So, all the patients in our study received TPO at a dosage of 300 U/kg from day +1 until platelet engraftment. GF is a serious complication in the patients with SAA post-HSCT. GF rates were 5–20% reported by previous studies, and the 1-year OS rate was only 30–40% (1, 23, 24). The age of patients over 40 years had a higher rate of GF (16). One patient (3.3%) in our study experienced PGF, and other 2 patients (6.7%) experienced SGF. Three patients died within 4 months after GF diagnosed. Two of them received donor PBSCs infusion and achieved engraftment again, but died of severe infection eventually. DSA is considered to be associated with increased risk of GF in HID-HSCT (25). The cutoff of mean fluorescence intensity (MFI) for treatment has not been consolidated. The desensitization regimen is consisted of plasma exchange with 1 volume of albumin exchange and immunoglobulin 500 mg/kg (days −14, −12, −10, −8, and −1), FK at 1 mg/kg or CsA at 3 mg/kg daily as continuous infusion, and MMF at 1 g twice a day (from day −14 to day −8) (26). Chang et al. reported that the usage of rituximab 375 mg/m^2^ in patients with DSA positive (2,000 ≤ MFI <10,000) could effectively prevent the onset of PGF (27). In our study, DSA in one patient showed positive MFI (8,806.43), and we used immunoglobulin and CsA at the above-mentioned dosage. The patient achieved myeloid and platelet engraftment successfully. Cy combined ATG has been a standard conditioning regimen in MSD-HSCT for SAA. The regimen has been modified in recent years. The addition of fludarabine in the condition regimen could reduce the dose of cyclophosphamide, reduce the toxic and side effects of drugs, and enhance immunosuppressive effect which is better for engraftment. Busulfan could also facilitate the engraftment for patients with active focal hyperplasia (28). We added busulfan to conditioning regimen in HID-HSCT according to “Beijing Protocol.” The detailed protocol included an intensified regimen that added busulfan (3.2 mg/kg/day administered iv. in divided doses on days −7 and −6) to Cy + ATG (29, 30). The use of high dose Cy post-HSCT has also been recommended by the EBMT SAAWP protocol in order to reduce GF (5). By far, no optimal conditioning regimen has been established for SAA, and it has been suggested to select a conditioning regimen according to patients' age, course of the disease, and transfusions in order to improve outcomes of HSCT.

In early studies, the incidence of severe aGvHD was relatively high post-HSCT, especially in HID-HSCT, and became an important factor that led to TRM (31). In recent years, immunosuppressive agents such as MTX, CsA, and MMF have been used more standardized, and conditioning regimen has been improved, the incidence of severe aGvHD has been sharply declined. Xu et al. (32) reported the outcomes of the patients with SAA who received HID-HSCT. The incidence of grade II–IV aGvHD was 20%, and the cumulative incidence of cGvHD at 3 years post-HSCT was 25.9%. Zhang et al. (7) compared the rates of aGvHD between HID-HSCT and MSD-HSCT. They found that the incidence of grade II–IV aGvHD in HID cohort was higher than that in the MSD cohort (21.4 vs. 5.3%), but no significant difference in grade III–IV aGvHD was observed between the 2 cohorts. In our study, there were no differences in cumulative incidences of grade II–IV and grade III–IV aGvHD between HID and MSD cohort, and no patient died because of severe aGvHD. No significant difference in cGvHD was found between the two cohorts. Though two patients in HID cohort experienced extensive cGvHD, no patient died due to cGvHD.

The incidence of infection increased in HID-HSCT due to early delayed immune reconstitution (33). CMV infection is one of the life-threatening complications after allo-HSCT. Uncontrolled CMV infection may cause CMV disease. In our cohort, the high incidence of CMV infection and diseases may be attributed to the high dosage of ATG (10 mg/kg). But it is controversial whether CMV infection incidence in patients with HID-HSCT is higher than that of MSD-HSCT. Zeng et al. (16) reported that patients received HID-HSCT had a higher rate of bacterial and fungal infection (68.2 vs. 36.7%), but no difference in CMV infection was observed between HID and MSD group (23.6 vs. 14.7%, *p* = 0.267). Zhang et al. (7) reported a different result. In their study, the incidence of CMV infection in HID group was higher than that in MSD group (80.0 vs. 55.3%, *p* = 0.046). In our study, the cumulative incidence of bacterial and fungal infection at 1 year was 72.7% in HID cohort and 37.5% in MSD cohort (*p* = 0.047), and the cumulative incidence of CMV infection at 3 years post-HSCT was 77.3 and 37.5% in HID and MSD cohort, respectively (*p* = 0.043).

In our center, we administered ganciclovir iv. at a dosage of 5 mg/kg two times a day between days −9 and −2 during conditioning to prevent CMV infection, and CMV PCR was monitored once a week for all the patients. If patients' CMV-DNA showed positive, ganciclovir (at a dosage of 5 mg/kg every 12 h daily) or trisodium phosphonoformate (at a dosage of 60 mg/kg every 8 h daily) would be administered iv. as preemptive treatment for 2–3 weeks according to the results of CMV-DNA. Routine CMV-DNA monitoring and preemptive ganciclovir or trisodium phosphonoformate treatment reduced early CMV disease in our cohort, but late CMV disease has become a threat to long-term survival. Studies on novel antivirus agents have been reported in recent years. It was reported in a phase 3 prophylaxis study that letermovir for 14 weeks after HSCT can significantly reduce the incidence of CMV infection, and it has been approved to be used for the prevention of CMV infection and disease (34). Other novel antivirus agents, such as maribavir and brincidofovir, also showed optimistic results in phase 2 clinical trials, but no significant reduction of CMV infection and disease was seen in phase 3 studies (35). In our study, five patients in HID cohort progressed to CMV end-organ disease. But after local or systemic administration of ganciclovir, no patient died because of CMV end-organ disease. We considered that by the measures of detecting CMV-DNA for donors before HSCT, using ganciclovir during conditioning, detecting CMV-DNA for the patients regularly after HSCT, and administering antiviral drugs on time, CMV infection may be under controlled. Therefore, the risk of death caused by CMV infection could be greatly reduced after HSCT. If CMV infection or disease cannot be controlled by conventional antiviral drugs, novel agents may be tried.

Our analysis had several limitations. First, the sample size of our study was relatively small. Age has been considered as a factor affected survival outcomes, but due to limited numbers of the patients, we did not analyze the outcomes by age subgroups. Second, we failed to acquire some patients' initial data such as transfusion units and level of ferritin, so we did not find any risk factors that affect OS, FFS, or GvHD. We will enlarge the sample size for further analysis in future. Despite these limitations mentioned above, our study showed comparable outcomes of HID-HSCT with MSD-HSCT for the patients of SAA.

In conclusion, the results in our research suggest that HID-HSCT is encouraging in survival rates and complications, and it is comparable to MSD-HSCT, especially in patients under 40 years. For the patients of SAA without MSD or URD, HID could be an alternative option.

## Data Availability Statement

The original contributions presented in the study are included in the article/supplementary materials, further inquiries can be directed to the corresponding author/s.

## Author Contributions

QingYuW: collected the data, performed the statistical analysis, and wrote the manuscript. YL and YD: conceived and designed the study, reviewed the manuscript, and provided guidance. HR, ZL, WL, YY, QingYaW, and QianW: collected the data and reviewed the manuscript. YS, WX, ZQ, JO, NH, and JW: followed-up the outpatients. All authors contributed to the study design and approved the final manuscript.

## Funding

This study was supported by the Youth clinical research project of the Peking University First Hospital (2018CR10) and the Science and Technology Innovative Cultivation Youth Foundation of Peking University (BMU2020PY007).

## Conflict of Interest

The authors declare that the research was conducted in the absence of any commercial or financial relationships that could be construed as a potential conflict of interest.

## Publisher's Note

All claims expressed in this article are solely those of the authors and do not necessarily represent those of their affiliated organizations, or those of the publisher, the editors and the reviewers. Any product that may be evaluated in this article, or claim that may be made by its manufacturer, is not guaranteed or endorsed by the publisher.
